# Measuring sexual dimorphism in human faces

**DOI:** 10.1111/joa.70056

**Published:** 2025-10-16

**Authors:** Cassidy Da Silva, Hanne Hoskens, J. David Aponte, Katherine Caine, Seth M. Weinberg, Peter Claes, Benedikt Hallgrímsson

**Affiliations:** ^1^ Department of Cell Biology and Anatomy University of Calgary Calgary Alberta Canada; ^2^ Center for Craniofacial and Dental Genetics, Department of Oral and Craniofacial Sciences University of Pittsburgh Pittsburgh Pennsylvania USA; ^3^ Department of Human Genetics KU Leuven Leuven Belgium; ^4^ Department of Electrical Engineering, ESAT/PSI KU Leuven Leuven Belgium

**Keywords:** 3D, CVA, facial sexual dimorphism, femininity, masculinity, regression scores

## Abstract

Facial shape is one of the most widely studied sexually dimorphic traits in humans. Sexually dimorphic facial shape has been linked to processes in neurodevelopment, immunocompetence, social perception, and mate preference. However, research into these associations has produced conflicting results, owing in part to the diverse methods used to quantify sexual dimorphism of the face. Our study compares two commonly used methods for measuring morphological sexual dimorphism: regression scoring and Canonical Variates Analysis (CVA; or linear discriminant analysis). We test both methods on a large sample of adult males (*n* = 540) and females (*n* = 540) with three‐dimensional (3D) descriptions of the whole face, both with and without prior decomposition of the allometric component. Our results show that CVA outperforms regression scoring, resulting in scores that are more accurate in classifying the sexes and recreating the male–female shape axis (i.e., the difference in shape means based on reported sex). We also find that height is positively associated with regression scores after controlling for sex (*p* < 0.01), but not with CVA scores. These results suggest the need for a possible reassessment of previous claims that taller males have more male‐like facial shapes, as well as a broader re‐evaluation of the literature that considers the significance of method selection in shaping research outcomes. We establish a foundation for more accurate comparisons of facial sexual dimorphism and its relationship to various domains of human health and biology.

## Graphical abstract

CVA outperforms regression scoring when measuring sexual dimorphism in human faces, allowing for more accurate classification and shape reconstruction. Method selection has a significant impact on findings, necessitating a reevaluation of previous claims linking facial masculinity to traits such as height.

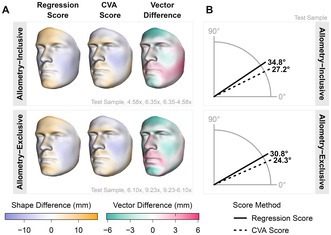

## 1 INTRODUCTION

Human faces are sexually dimorphic in shape, with differences between males and females extending to nearly every region of the face (Bannister et al., 2022; Matthews et al., 2023). Male faces tend to have more sloped foreheads, more prominent brows, larger noses, and wider chins, whereas female faces have more vertical foreheads, fuller cheeks, and tapered chins (Bannister et al., 2022). Within each sex, individuals vary in their expression of these sexually dimorphic traits; thus, facial sexual dimorphism is a continuous trait (Mitteroecker et al., 2015), with variations linked to processes in development (Tan et al., 2017; Whitehouse et al., 2015), immunocompetence (Boothroyd et al., 2013), social perception (Fiala et al., 2022; Rostovtseva et al., 2022), and mate preference (Munoz‐Reyes et al., 2015). However, studies of these associations have yielded conflicting results. This is likely due to differences in how facial sexual dimorphism is quantified, as well as the theoretical bases on which measures of dimorphism are premised (Mitteroecker et al., 2015; Sanchez‐Pages et al., 2014).

Various conceptualizations of facial sexual dimorphism have been proposed, including subjective ratings of perceived “masculinity” and “femininity”, as well as objective measurements of facial shape dimorphism (Mitteroecker et al., 2015). Perceived ratings typically have a low to moderate correlation with underlying shape differences between the sexes, and they are influenced by psychological and cultural factors (Boothroyd et al., 2013; Fiala et al., 2022; Gilani et al., 2014; Holzleitner et al., 2014; Kleisner et al., 2021; Koehler, Simmons, Rhodes, & Peters, 2004; Komori et al., 2011; Lee et al., 2014; Mitteroecker et al., 2015; Sanchez‐Pages et al., 2014). Therefore, a clear distinction must be made between subjective and objective concepts of facial sexual dimorphism, as they can differ significantly in terms of underlying mechanisms as well as implications for human biology and social perception. In this paper, we study the shape component of facial sexual dimorphism, which refers to the individual expression of traits that differ on average between male and female faces (Mitteroecker et al., 2015). While overall sexual dimorphism encompasses both size and shape variation, we focus on only shape. We use the terms *facial sexual dimorphism*, *maleness*, and *femaleness* (along with *male‐like* and *female‐like*) to refer to the expression of sex‐related shape differences once size variation is removed, whereas *masculinity* and *femininity* refer to observer ratings of sex likeness (Mitteroecker et al., 2015).

Researchers have used a wide range of techniques to capture facial sexual dimorphism in individual faces. One category of methods uses linear distances, angles, or ratios measured between a set of anatomical landmarks that differ significantly between males and females. The facial width‐to‐height ratio is by far the most commonly used measure in this category. Males are reported to have shorter midfaces relative to their facial width than females (Hodges‐Simeon et al., 2021; Weston et al., 2007), prompting some researchers to use facial width‐to‐height ratio as an indicator of increased facial maleness (Lefevre et al., 2014; Little et al., 2013; Wu et al., 2022). Other examples include the lower‐face‐to‐whole‐face‐height ratio, eye‐mouth‐eye angle, and cheek‐bone prominence (Little et al., 2008; Penton‐Voak et al., 2001; Wu et al., 2022). This approach has been expanded to composite indexes that combine values from multiple ratios or measures (Koehler, Simmons, & Rhodes, 2004; Koehler, Simmons, Rhodes, & Peters, 2004; Little et al., 2008; Pound et al., 2009; Scheib et al., 1999; Soler et al., 2012). For example, Scheib et al. (1999) developed a method that combined cheek‐bone prominence and relative lower facial height by converting both measurements to *Z*‐scores and adding them to produce a single index. While these methods can be applied to two‐dimensional or three‐dimensional data, they are most often used with two‐dimensional photographs of participant faces. Despite including multiple measures of facial sexual dimorphism, these scores only account for a fraction of the known sexually dimorphic facial traits in humans (Bannister et al., 2022; Matthews et al., 2023). The defining feature of this class of methods is that specific measures are premised on a prior expectation of which features possess the greatest sex differences.

A second category of methods attempts to capture sexual shape dimorphism of the whole face. These measures, which can be derived from sets of linear distances (Gilani et al., 2014), landmark configurations (Komori et al., 2011), or direct photographic inputs (Chen et al., 2020), aim to quantify facial sexual dimorphism without making a priori hypotheses about which features contribute most to male–female shape difference. Such approaches are required, for example, to determine how sexually dimorphic the face is and how the magnitude of facial sexual dimorphism variation relates quantitatively to individual, ontogenetic, or geographically patterned variation. Geometric morphometric analysis of two or three‐dimensional coordinates is a popular approach that falls into this category.

Geometric morphometric datasets can be analyzed in a variety of ways, and the choice of method can impact the quantification of facial sexual dimorphism (Hahn et al., 2019; Mitteroecker et al., 2015; Sanchez‐Pages et al., 2014). The most commonly used approaches are regression scoring (Danel et al., 2018; Fiala et al., 2022; Holzleitner et al., 2014; Kleisner et al., 2021; Komori et al., 2011; Marcinkowska & Holzleitner, 2020; Tan, Maybery, Ewing, et al., 2020; Valenzano et al., 2006; Zaidi et al., 2019) and Canonical Variates Analysis (CVA, also called linear discriminant analysis or LDA) (Best et al., 2018; Boothroyd et al., 2013; Lee et al., 2014; Munoz‐Reyes et al., 2015; Nila et al., 2019; Rostovtseva et al., 2022; Sanchez‐Pages et al., 2014; Scott et al., 2010; Van Dongen, 2014). The regression scoring method calculates facial sexual dimorphism as the individual positions along the male–female shape axis, i.e., the orthogonal projections of data points onto the vector spanning male and female shape means (Komori et al., 2011). In contrast, CVA calculates this axis in such a way that it maximizes the distance between group means (Zelditch et al., 2004). This is accomplished by rotating and rescaling the space to align with the within‐group pooled variances, i.e., multiplying the male–female shape axis by the inverse pooled within‐sex covariance matrix (Mitteroecker et al., 2015). Facial sexual dimorphism is then measured as the orthogonal projections of the data onto the first canonical variate axis (Scott et al., 2010; Tan et al., 2017). CVA and LDA are different terms for the same method of maximizing separation across two or more groups (Manthey & Ousley, 2020; Mitteroecker & Bookstein, 2011). Other methods within this category include Procrustes distance from the opposite‐sex mean (Sanchez‐Pages et al., 2014; Sanchez‐Pages & Turiegano, 2010, 2013), Random Forest classification (McKenna et al., 2021), and Convolutional Neural Networks (Chen et al., 2020). Despite this variety of methods, no studies have been conducted to objectively compare their relative accuracy. Such comparisons are necessary to make informed decisions about which method to use or reconcile conflicting literature on the role of facial sexual dimorphism in various aspects of human health and biology.

Height is an important covariate or confounding factor for the study of human facial sexual dimorphism, as males are typically taller, and facial shape correlates allometrically with height (Larson et al., 2018). Taller people tend to have larger, longer, and more prognathic faces (Larson et al., 2018; Mitteroecker et al., 2013). Because stature is also sexually dimorphic, the covariation of facial shape and height is thought to account for a portion of the shape differences observed between the sexes. Some studies of human facial sexual dimorphism exclude size‐related shape variation by decomposing the facial sexual dimorphism axis into its allometric and non‐allometric components (Kleisner et al., 2021; Mitteroecker et al., 2015; Zaidi et al., 2019). While the shape effects of these components have been previously described (Larson et al., 2018; Mitteroecker et al., 2013), we do not currently understand how the procedure of removing size‐related shape variation interacts with the different methods of scoring sexual dimorphism.

In this paper, we present a quantitative comparison of different techniques for measuring sexual dimorphism in human facial shape. We focused on regression scoring and CVA, which began with 3D descriptions of the whole face, both with and without the allometric component included. We evaluated these methods for measuring facial sexual dimorphism in two ways: (1) by determining how well the facial sexual dimorphism scores discriminate between the sexes in a classification setup, and (2) by measuring how well the male–female shape axis could be recreated by the scores.

## 2 METHODS

### 2.1 Sample description

The data for this project was sourced from the 3D Facial Norms (3DFN) project (Weinberg et al., 2016), part of the National Institute of Dental and Craniofacial Research's FaceBase data repository. Participants in this project were recruited from 2009 to 2014 in four US cities: Pittsburgh, Seattle, Houston, and Iowa City. Inclusion criteria required that participants must have reported no personal or family history of birth defects or syndromes affecting the head and/or face, no personal history of facial trauma and/or surgery, and no personal medical conditions that affect facial structure. Enrollment was also restricted to people currently living in the United States who self‐identified as White, non‐Hispanic, and of recent European ancestry (Weinberg et al., 2016). The 3D Facial Norms dataset included 3D facial images of 2454 participants: 952 males and 1502 females (ages 3–40 years old). All participants provided written informed consent prior to participating in the 3DFN study. The secondary use of the 3DFN dataset was approved under ethics standards by the University of Calgary Conjoint Health Research Ethics Board (REB15‐1342).

### 2.2 3D phenotyping

Participants' facial surfaces were captured using the two‐pod 3dMDface™ system or the multi‐pod 3dMDcranial™ system (Weinberg et al., 2016).

The MeshMonk toolbox is a set of tools designed for high‐throughput phenotyping of 3D biological images, which generate dense meshes of landmarks across image surfaces. We used the MeshMonk toolbox (White et al., 2019) in Matlab (The MathWorks Inc., 2023) to fit an atlas of 5629 landmarks (each with x, y, and z coordinates) to each scan so that participants were described by the same number of uniformly positioned points, also known as “quasi‐landmarks” (White et al., 2019). We manually placed five guide points (glabella, pronasale, pogonion, left tragion, and right tragion) to facilitate scaling, translation, and rotation of the atlas onto the target in the first rigid registration step, followed by a non‐rigid transformation step where the atlas was warped to fit each participant's facial surface (White et al., 2019). The procedure was repeated for the bilaterally reflected copies of the original images. Registrations from the original and reflected images were imported into R (R Core Team, 2024), where they were superimposed (Schlager, 2017) and averaged to produce symmetric representations of the participants' faces.

### 2.3 Data processing

We manually reviewed each raw scan and excluded any images with missing anatomy, closed eyes, open mouths, facial expressions, and/or facial hair. Following MeshMonk registration, 3D surfaces were reviewed again for possible registration errors. Possible errors were identified using Procrustes distance and the percentage of correspondence outliers reported by MeshMonk toolbox (White et al., 2019). Participants who were more than one standard deviation from the mean and/or possessed ≥10% correspondence outliers (indicating poor scan quality) were manually screened for errors and excluded when necessary. We also excluded participants who were under the age of 18, related to other participants in the sample, or missing information about their age, sex, height, or weight. Finally, we identified and excluded height outliers (i.e., recording errors) by regressing height as a function of weight and visually inspecting the facial scans. Following all exclusions, the sample consisted of 1671 adults (542 males and 1129 females) for further study. To obtain a balanced sample of participants from both sexes, we randomly sampled 540 males and 540 age‐matched females for the analysis (M = 26.1, SD = 5.3, Figure S1).

To ensure an unbiased evaluation of the different scoring techniques, we further divided our sample into age‐ and sex‐matched training and test sets. In Figure S2, we used a permutation test to investigate how the direction of the male–female shape vector changed with different sample sizes. We iteratively sampled pairs of male and female age‐matched participants, computed the male–female shape vector, and compared it to the overall dataset's (*N*_total_ = 1080) male–female shape vector using the vector angle as a measure of similarity. Using this approach, we determined that vector differences started to plateau at 300 participants and divided our sample accordingly (*N*_training_ = 780, *N*_test_ = 300).

To prevent the test sample from influencing the training sample models (i.e., data leakage), processing steps were carried out sequentially, with the training sample processed first and the test sample processed to match. We used General Procrustes analysis to superimpose and scale the training data to a unit size (Schlager, 2017). Next, Principal Component Analysis (PCA) was used to reduce the dimensionality of the facial shape data and thus the possibility of overfitting. We applied PCA to the training sample, followed by parallel analysis (Blighe & Lun, 2020), to determine the number of informative principal components to keep. In this analysis, we used the first 36 principal components, which accounted for 93.7% of the total variation in facial shape in the training sample. Finally, we obtained the non‐allometric shape components by taking the residuals of a height‐on‐shape regression, where shapes were represented by the principal component scores. Figure S3 shows the distribution of heights in the training sample, stratified by sex. Correspondingly, the test data was aligned with the training configurations and projected into the PCA space by subtracting the training mean and multiplying the residuals by the 36 training‐derived eigenvectors. Finally, we calculated the test sample residuals using the height‐on‐shape model described previously.

### 2.4 Sexual dimorphism scoring

Test sample participants were assessed for facial sexual dimorphism using two methods: regression scoring and CVA. Regression scores were calculated by orthogonally projecting each subject onto the sex coefficients from a sex‐on‐shape regression model, i.e., the regression vector spanning male and female shape means in the training sample (Drake & Klingenberg, 2008). CVA was implemented with the Morpho package (Schlager, 2017). Both scoring procedures were used on the principal component scores, with and without prior removal of the allometric component. Furthermore, both methods used the training set to create sexual dimorphism models (sex regression vector or CVA model) that were used to score the smaller test set. To facilitate comparisons, CVA and regression scores were converted to *Z*‐scores prior to analysis. Faces scored 0 represent the midpoint between male and female shape means (i.e., the sample average), whereas positive scores indicate a face closer to the male mean and negative scores indicate a face closer to the female mean.

### 2.5 Effects of sex on shape

We conducted a Procrustes ANOVA using the procD.lm function from geomorph to analyze the relationship between facial shape, sex, height, and their interaction in the training sample (Adams & Otárola‐Castillo, 2013; Baken et al., 2021). We excluded the interaction term if it was not significant.

Next, we visualized the shape effects of sex using the sex‐on‐shape regression vectors described above, which represent the allometry‐inclusive (“total”) and allometry‐exclusive (“non‐allometric”) sexual dimorphism axes established by the training dataset. We then used heatmaps to visually compare sex‐shape effects with and without the allometric component. Heatmaps were created using the Morpho package by plotting the closest point distance between target and reference surface meshes (Schlager, 2017).

We used a data‐driven clustering approach to segment the facial surface and analyze localized sex differences (Claes et al., 2018; Huang et al., 2021; Matthews et al., 2023). Clustering analyses were conducted on a randomly sampled age and sex‐matched cohort from the 3FDN dataset. Prior to analysis, sample shape coordinates were corrected for the effects of age, age^2^, sex, height, and weight. After obtaining the corrected shape variables, we calculated pairwise RV coefficients between landmark coordinates to create a square matrix of 3D correlations (Huang et al., 2021). We then used a PCA to extract the first ten principal components from the correlation matrix. Finally, we used silhouette analysis to calculate the optimal number of clusters (*k* = 9) and *k*‐means clustering to define the facial segments. The facial segments were bilaterally symmetric and followed well‐known anatomical regions of the face, such as the (1) frontal, (2) temporal and zygomatic, (3) orbital, (4) infraorbital, (5) nasal root, (6) nasal, (7) oral, (8) buccal and parotid, and (9) mental regions (Figure S4). We then applied this segmentation pattern to the training sample, dividing participants' facial surfaces into individually analyzable collections of facial landmarks. Each group of segments (e.g., training sample mouths) was individually aligned using Procrustes superimposition and then analyzed using a sex‐on‐shape regression. We calculated the coefficient of determination (*R* ^2^) for each segment, which represents the percentage of shape variation in the segment explained by sex. The results were visualized in a heatmap.

We also examined the separation of sex on the major patterns of shape variation described by the training sample eigenvectors. For each principal component, we used a Pearson correlation coefficient to calculate the relationship between the principal component scores and sex in the training dataset; a Bonferroni correction was then applied to determine the components with the strongest correlations.

### 2.6 Comparing facial sexual dimorphism scores

The facial sexual dimorphism scores from the test sample were evaluated in two ways: (1) by determining how well the generated scores discriminate between the sexes in a classification setup, and (2) by measuring how well the male–female shape axis could be recreated using the scores. Score classification ability was assessed by comparing Pearson correlation coefficients with sex, receiver operating characteristic (ROC) curves, and cross‐validated classification accuracy. The ROC curves plotted the true positive fraction against the false positive fraction across varying decision thresholds. We used the area under the curve (AUC) to quantify the models' overall abilities to distinguish between male and female faces, where larger areas indicated better classification performance (Alpaydin, 2020). We also calculated the 10‐fold cross‐validated classification accuracy of each scoring method using a general linear model implemented by the caret package (Kuhn, 2008).

Next, we used linear regression to model the effects of the facial sexual dimorphism scores on facial shape, while correcting for sex (i.e., shape ~ sex + score). The resulting regression vectors were scaled to a unit size and compared to the male–female shape axis using the vector angle, where smaller angles indicated greater similarity. The male–female shape axis was calculated as the difference in shape means based on reported sex in the training sample. We used heatmaps to visually compare score regression vectors. Additionally, we investigated individuals who scored closer to the female mean in one method (<0) and closer to the male mean in the other (>0), allowing us to evaluate where the two scores diverged in their assessments of facial sexual dimorphism. Equal samples were taken from all four quadrants (*N* = 14; i.e., maximum sample size given the available data), and their mean shapes were calculated. Finally, we used the same procedure as for sex (c.f., Effects of Sex on Shape) to investigate the separation of sexual dimorphism scores onto the eigenvectors, as well as linear regression to compare the scores' associations with height.

### 2.7 3D morphs

For various analyses, we created scaled (i.e., exaggerated) 3D morphs to aid in shape visualization. Paired morphs were generated by adding and subtracting a scaled shape vector from the sample mean. Following Matthews et al. (2023), we experimented and determined that scaling vectors to a Procrustes distance of 0.1 resulted in morphs with visible shape differences. As a result, scaled morphs are used solely to visually compare shape direction, rather than magnitude. Prior to plotting, morphs were multiplied by the mean centroid size to report results in recognizable units (mm).

### 2.8 Data availability and code

All code used to perform analyses and create figures is included in the Data S1 and S2. 3D facial surface models are available for the 3D Facial Norms dataset through the FaceBase Consortium (http://www.facebase.org/) at accession FB00000491.01 (DOI: https://doi.org/10.25550/VWP).

## 3 RESULTS

**FIGURE 1:**
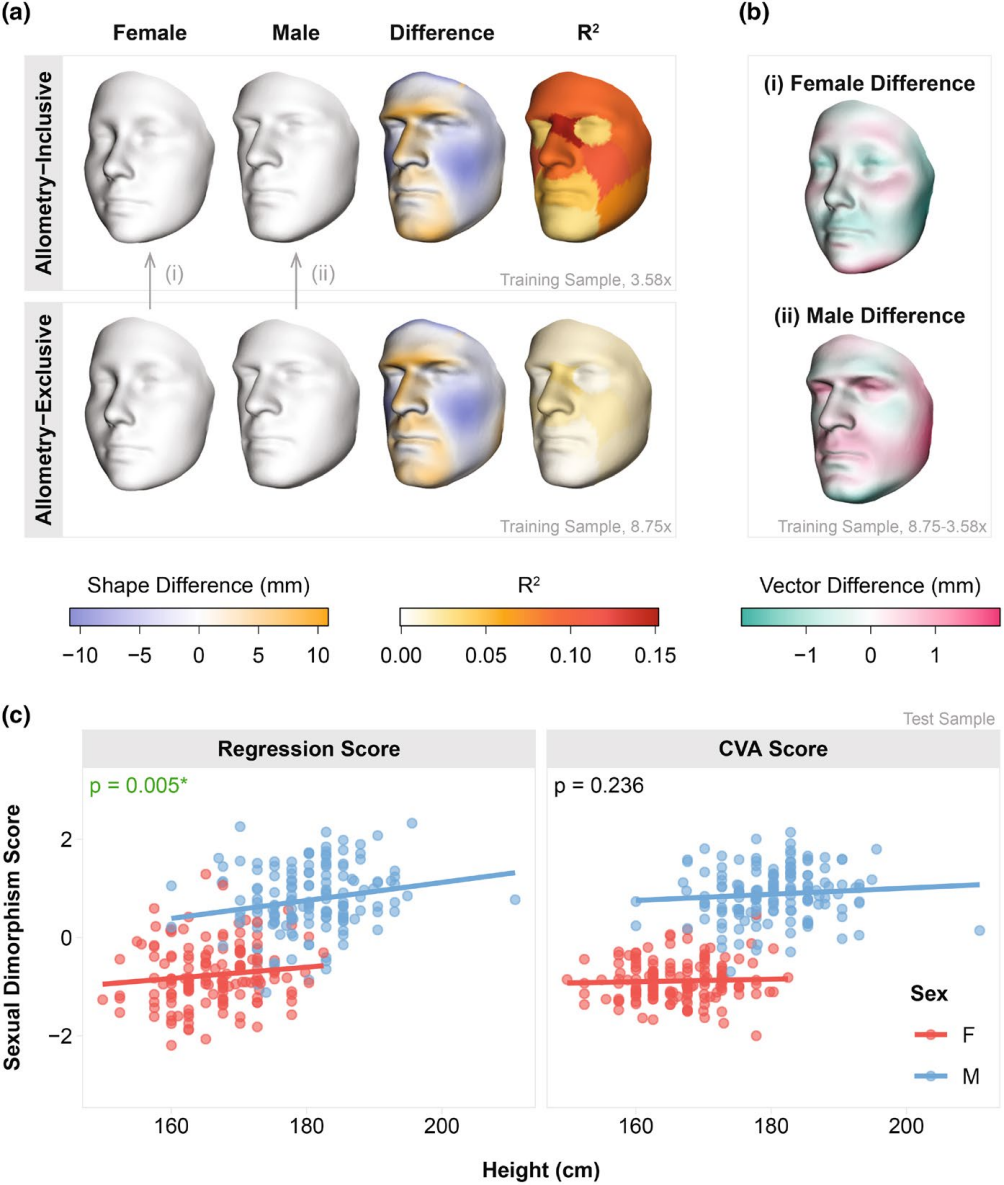
Allometry‐inclusive and allometry‐exclusive facial sexual dimorphism. (a) Scaled 3D morphs showing the effects of sex on shape in the training sample, both with (3.58×) and without (8.75×) the allometric component. Exaggerated male and female morphs from the training sample are shown alongside heatmaps indicating the differences between them (i.e., how the male morph differs from the female morph). An additional heatmap shows the distribution of *R* ^2^ values across facial segments in the training sample, with higher values indicating a greater proportion of shape variation explained by sex. (b) Heatmap showing how the non‐allometric component of sex differs from the allometry‐inclusive sex vector, (i) in females and (ii) males from the training sample. (c) Scatterplots showing the relationship between height and facial sexual dimorphism scores in the test sample (i.e., score ~ sex + height); *p*‐values correspond to the significance of height when controlling for sex (Type III Sums of Squares).

The difference between male and female shape means was statistically significant, with sex accounting for 3.5% (*p* < 0.001) of the total shape variation and 1.7% (*p* < 0.001) of the non‐allometric shape variation in the training sample (Tables S1 and S2). Compared to females, males had more oblique (i.e., sloped backwards) foreheads, more prominent supraorbital rims and glabellas, wider and larger noses, flatter cheeks, greater projection of the oral and mental regions, and more angular jawlines (Figure 1a, Video S1). The cheeks, nose, and brow showed the most significant sex differences, indicated by higher *R* ^2^ values, with the nasal root showing the greatest sex difference.

The direction of sexual shape dimorphism was very subtly different between shapes with and without the allometric component: after decomposing the allometric component, males had less projection in their cheeks, chin, and outer brow, combined with greater projection in the philtrum and upper lip (Figure 1b). In contrast, the removal of allometric variation had a significant impact on the magnitude of sexual dimorphism; shapes that retained allometric variation showed much clearer and stronger dimorphisms overall (Figure S5). To better understand the role of height in male–female shape differences, we used linear regression to examine the correlation between height and allometry‐inclusive sexual dimorphism scores in the test sample. Height was positively associated with regression scores while controlling for sex (*p* < 0.01), but the relationship was not significant when CVA scores were used (Figure 1c).

**FIGURE 2:**
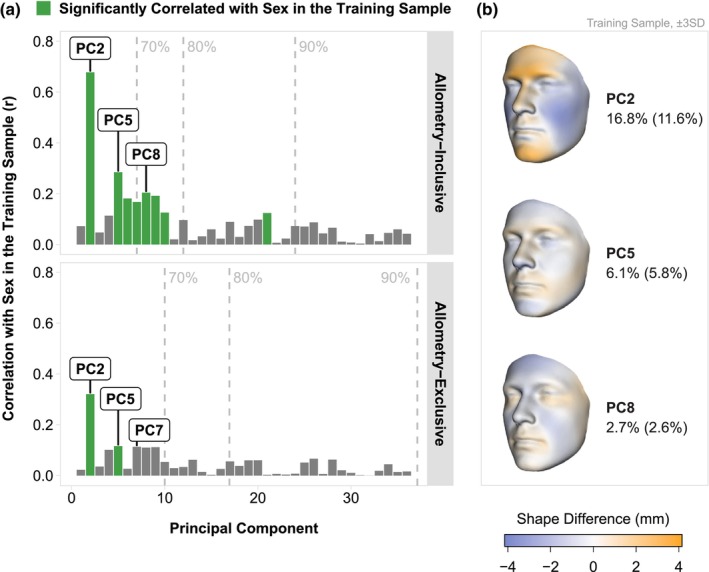
Relationship between principal components and sex in the training sample. (a) Barplots depicting the correlation of principal component scores with sex in the training sample; bars in green are significantly correlated with sex after Bonferroni multiple testing correction for 36 principal components (*p* < 0.0014); dashed lines indicate the number of components explaining 70%, 80%, or 90% of the total shape variation prior to removal of allometric variation, where the 36 non‐allometric components cumulatively explain 87.5% of the total shape variation. (b) 3D heatmaps of shape change in three components (±3SD) with the highest correlation with sex: principal components two, five, and eight. Percents of total shape variation explained are displayed beside the heatmaps before and after removal of the allometric variation (in parentheses).

During our PCA, we identified several eigenvectors with significant sex associations in the training sample (Figure 2). After multiple testing correction, eight principal components from allometry‐inclusive shapes and two components from allometry‐exclusive shapes showed a significant correlation with sex (Figure 2a). Several of the most strongly correlated components, such as principal components two, five, and eight, represent different aspects of male–female shape difference. The second eigenvector was the most highly correlated with sex, exhibiting a wide range of sexually dimorphic characteristics such as a prominent brow, nose, and chin combined with reduced cheeks (Figure 2b). Later components, such as components five and eight, each have a smaller fraction of the known sexually dimorphic features, like a pronounced brow or reduced cheeks, respectively.

**FIGURE 3:**
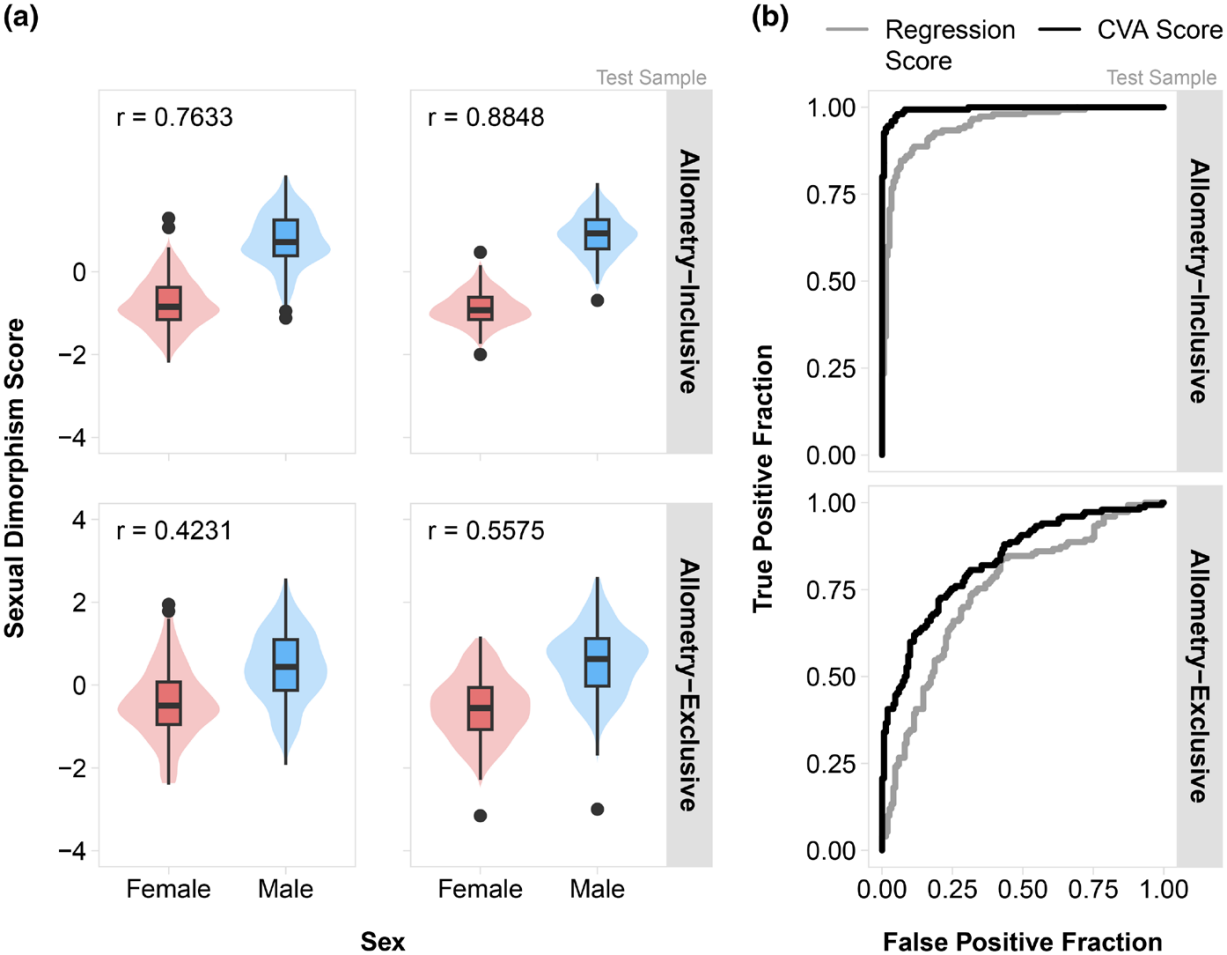
Classification accuracy of facial sexual dimorphism scores by method in the test sample. (a) Violin plots depicting the density of facial sexual dimorphism scores in the test sample, with a boxplot overlay showing the interquartile range (box limits) and median score by sex (bold line); whiskers extend up to 1.5 times the interquartile range, with participants outside of this range represented by individual data points. Pearson correlation coefficients of facial sexual dimorphism scores with sex are also shown (i.e., score ~ sex, Type III Sums of Squares). (b) ROC curves showing the overall diagnostic accuracy of facial sexual dimorphism scores in the test sample.

Figure 3 shows the results of the classification accuracy analysis in the test sample. Regression scoring and CVA produced varying degrees of score separation between males and females. In both allometry‐inclusive and allometry‐exclusive analyses, CVA scores correlated more strongly with sex than regression scores (Figure 3a). CVA scores were also consistently better sex classifiers; both ROC curves showed that CVA scores had a greater area under the curve (AUC) than regression scores, indicating a greater ability to distinguish between male and female faces (Figure 3b, Table 1). CVA scores also exhibited better classification accuracy in the 10‐fold cross‐validation analyses (Table 2). As expected, allometry‐inclusive scores outperformed allometry‐exclusive scores, as more sex variation was retained for classification.

**TABLE 1:** Area under the curve (AUC) for ROC plots of facial sexual dimorphism scores by method.

	Sexual dimorphism score	AUC
Allometry‐Inclusive	Regression Score	0.95
	CVA Score	0.99
Allometry‐Exclusive	Regression Score	0.75
	CVA Score	0.83

**TABLE 2:** 10‐fold cross‐validation accuracy measurements of facial sexual dimorphism scores by method.

	Sexual dimorphism score	Accuracy
Allometry‐Inclusive	Regression Score	86% ± 0.04%
	CVA Score	96% ± 0.04%
Allometry‐Exclusive	Regression Score	69% ± 0.11%
	CVA Score	75% ± 0.07%

**FIGURE 4:**
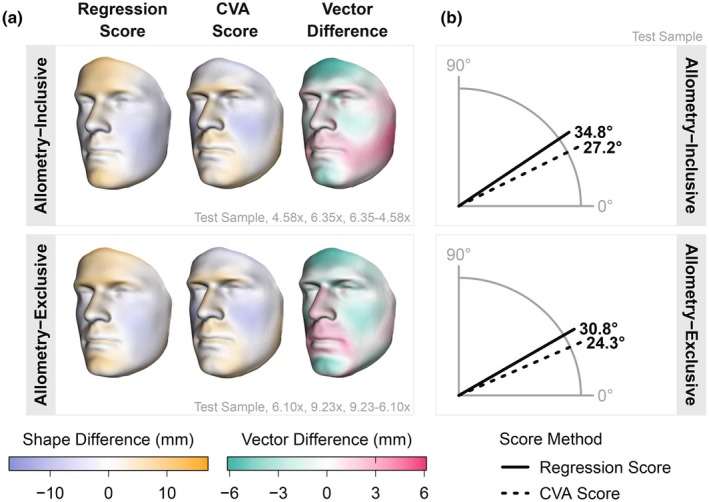
Facial sexual dimorphism score shape effects and reproduction of the male–female shape axis. (a) Scaled heatmaps of the regression (4.58×) and CVA score (6.35×) shape effects. Shape effects were modelled in the test sample and represent the effect of sex on shape, while controlling for sex (i.e., shape ~ sex + score). We also include heatmaps showing their vector differences; specifically, vector differences show how the CVA score shape effect differs from the regression score shape vector. (b) Angle plots show the relationship between score shape effects modelled in the test sample and the male–female shape axis established by the training data.

In Figure 4, we modelled the shape effects of regression and CVA scores when controlling for sex (i.e., shape ~ sex + score), as well as compared them to the male–female shape axis, i.e., the vector spanning male and female shape means established by the training sample. Regression and CVA score shape effects were visibly different, with the greatest disparities concentrated in the forehead region. Compared to regression scores, CVA scores captured male shapes with less forehead, cheek, and chin projection, as well as more robust and angled jawlines (Figure 4a). In comparison, male shapes captured by the regression score had a distinct forehead phenotype, in which the projection of the brow extended upward to encompass the entire forehead. This forehead phenotype did not appear in either the allometry‐inclusive or allometry‐exclusive male–female shape vectors; however, it strongly resembled the shape effect from the second eigenvector (Figures 1a and 2b). Furthermore, comparing the vector angles revealed that CVA score shape effects were more similar to the male–female shape axis than regression score shape effects (Figure 4b).

In addition to direct comparison of vector differences, we also investigated individuals who scored closer to the female mean in one method (<0) but closer to the male mean in the other (>0), or vice versa. We took equal samples (*N* = 14) from both of the above groups and calculated their mean shapes, which revealed similar shape differences concentrated around the forehead and chin (Figure S6). Notably, both samples largely consisted of males and females who had been misclassified by the regression score: females who were projected closer to the male mean (>0) had greater forehead and chin projection compared to the test sample average, while males who were projected closer to the female mean (<0) had reduced projection in those same areas. Regression and CVA scores differed similarly between allometry‐inclusive and allometry‐exclusive analyses; however, the differences between methods were greater in the non‐allometric facial sexual dimorphism scores (Figures 4 and S6).

**FIGURE 5:**
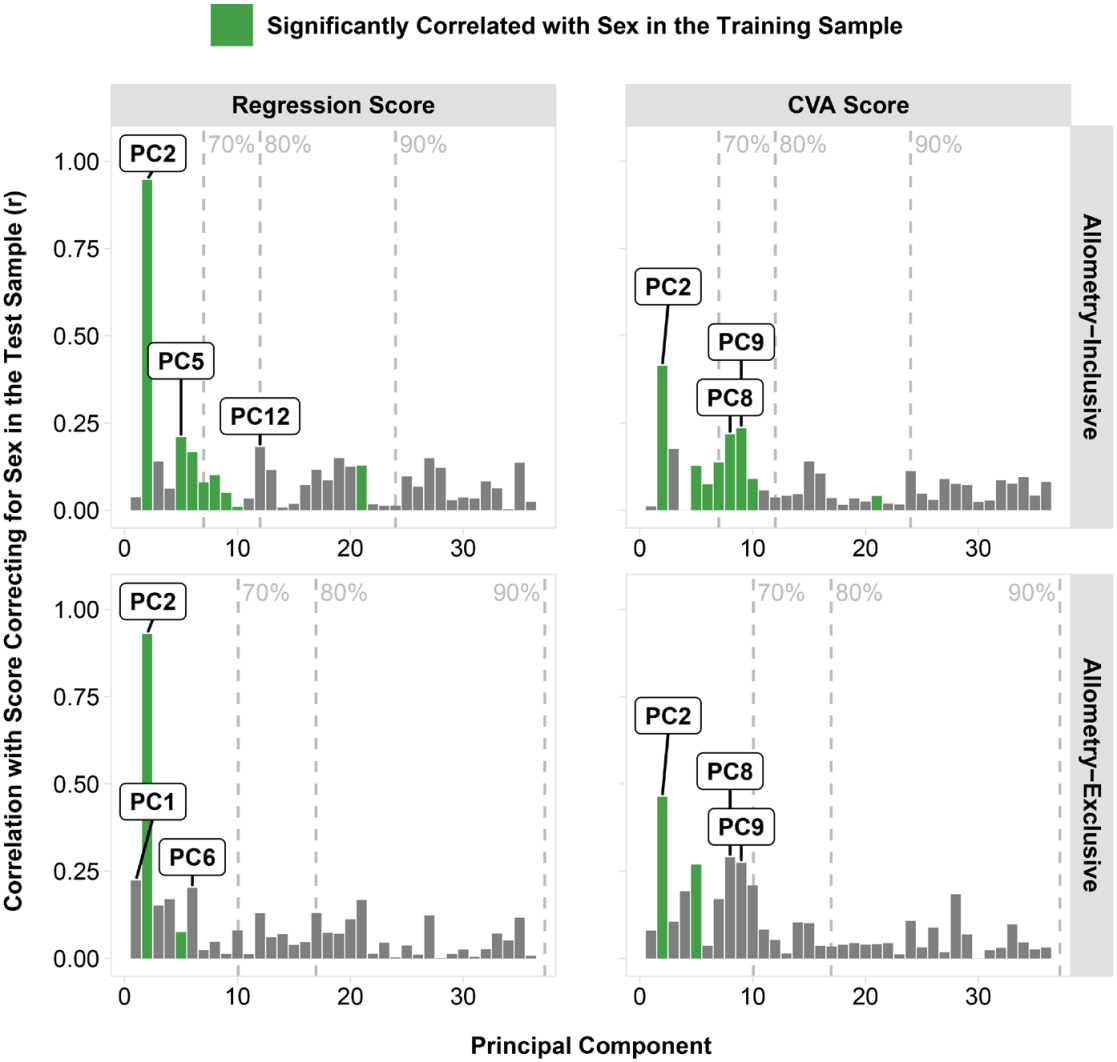
Bar plots showing the relationship between principal components and facial sexual dimorphism scores. The green bars indicate components that are significantly correlated with sex in the training sample after Bonferroni multiple testing correction for 36 principal components (*p* < 0.0014). The labels identify the top three components correlated with facial sexual dimorphism scores, while controlling for sex, in each panel. The dashed lines indicate the number of components explaining 70, 80, or 90% of the total shape variation prior to removal of allometric variation, where the 36 non‐allometric components cumulatively explain 87.5% of the total shape variation.

Given the strong resemblance between the regression score shape effects and the second eigenvector, we wanted to investigate further. We followed the same procedure as with sex to examine the relationship between the sexual dimorphism scores and the principal components; the results are shown in Figure 5. In both allometry‐inclusive and allometry‐exclusive analyses, regression scores were strongly correlated with the second principal component (*r* > 0.9) and weakly correlated with the others. In contrast, CVA scores were moderately to strongly correlated with several components, the majority of which had previously been identified as being strongly correlated with sex (e.g., principal components two, five, and eight; Figure 2). Figure S7 compares the sex and score correlations. While CVA scores followed an approximately linear relationship between sex and score correlations, regression scores exhibited an exponential relationship, with the second principal component leading the trend. The rate of increase, or the “steepness” of the curve, was greater in non‐allometric regression scores.

## 4 DISCUSSION

Sexual selection is the most commonly cited explanation for facial sexual dimorphism in humans. Within this framework, researchers have proposed various selective benefits that may be signaled by facial sexual dimorphism, mediated by the masculinizing or feminizing effects of testosterone and estrogen, respectively. Attraction to testosterone‐dependent traits is thought to be an evolutionary adaptation that helps females find mates who either benefit them directly or benefit their offspring through the transmission of “good” genes (Andersson, 1994; Zaidi et al., 2019). The assumption that increased facial maleness signals attractive qualities in males is largely driven by proponents of the immunocompetence handicap hypothesis, which states that facial maleness is a sexual ornament that indicates higher immunocompetence in males. Testosterone has been shown to suppress immune activity in humans (Trigunaite et al., 2015), prompting researchers to consider a possible trade‐off between immune function and the development of male ornaments (Folstad & Karter, 1992). According to the immunocompetence handicap hypothesis, males with better immune function can tolerate higher levels of testosterone and thus produce more extravagant sexually dimorphic traits (Folstad & Karter, 1992). However, the literature on the immunocompetence handicap hypothesis in humans is mixed, with some studies finding an association (Moore et al., 2011; Rantala et al., 2012) and others not (Nowak et al., 2018; Scott et al., 2013; Zaidi et al., 2019). This is due in part to the diversity of methods used to quantify sexual dimorphism of the face.

Developing an accurate measure of facial sexual dimorphism may also be of clinical interest, as facial maleness has been linked to prenatal testosterone exposure and a variety of neurodevelopmental disorders, including autism spectrum disorder, attention deficit disorder, and intellectual disability (McKenna et al., 2021; Tan et al., 2017, 2022; Tan, Maybery, Ewing, et al., 2020; Tan, Maybery, Gilani, et al., 2020). The link between facial maleness and autism‐related traits and behaviors has been found in a variety of cohorts, including autistic children, their parents, and healthy control groups (McKenna et al., 2021; Tan et al., 2017, 2022; Tan, Maybery, Ewing, et al., 2020; Tan, Maybery, Gilani, et al., 2020). Thus, an effective measure of facial sexual dimorphism may serve as a potential indicator of fetal testosterone exposure, disease risk, and disease severity in these conditions (McKenna et al., 2021).

Researchers have used a variety of methods to measure facial sexual dimorphism, ranging from simple linear measurements to complex multivariate evaluations of facial shape using geometric morphometrics. While some researchers have observed differences in scores produced by diverse methods (Hahn et al., 2019; Mitteroecker et al., 2015; Sanchez‐Pages et al., 2014), the underlying causes and consequences of these differences have not been investigated further. To our knowledge, no prior studies have objectively compared the relative performance of the various ways of measuring sexual dimorphism of the face. Such comparisons are necessary to make informed decisions about which methods to use and so would be of great practical utility in this area of research. In this paper, we compared the two most common methods used in the literature, regression scoring and CVA, which began with 3D descriptions of the face, both with and without the allometric component.

Sexual dimorphism in the 3DFN dataset has been previously described elsewhere (Bannister et al., 2022; Kesterke et al., 2016; Matthews et al., 2023). In our study, we found that sex differences in our training sample were consistent with those previously reported. On average, males had more oblique foreheads, more prominent brows, larger noses, and wider chins, whereas females had more vertical foreheads, fuller cheeks, and more tapered chins. Sex differences were only subtly different between shapes with and without the allometric component; after decomposing the allometric component, males had less projection in their cheeks, chin, and outer brow, combined with greater projection in the philtrum and upper lip. While few studies have explicitly compared allometry‐inclusive (i.e., “total”) and non‐allometric sexual dimorphism, a visual inspection of morphs created by other researchers suggests only minor differences (Fiala et al., 2022; Mitteroecker et al., 2013).

The most notable difference between allometry‐inclusive and allometry‐exclusive shapes was the magnitude of sex‐shape variation. Since males are taller on average, height is an important confounding factor in the study of facial sexual shape dimorphism; standardizing for one variable inadvertently excludes some of the variation associated with the other. As expected, allometry‐inclusive shapes were more dimorphic and outperformed allometry‐exclusive shapes in a classification setup, as more sex variation was retained for classification.

The covariation of facial shape and height is thought to account for a portion of the morphological differences observed between the sexes. Previous studies have noted significant associations between increased height and higher levels of both perceived masculinity (Holzleitner et al., 2014; Mitteroecker et al., 2015) and quantitative measures of facial maleness using regression scoring (Zaidi et al., 2019). We also discovered a small but significant relationship between sexual dimorphism regression scores and height; however, this association was not significant for CVA scores. These results may suggest the need for a reassessment of previous claims that taller males have more male‐like facial shapes, as well as a broader re‐evaluation of the literature that considers the various methods used to quantify facial sexual dimorphism of the face. Depending on the method used, these results also raise the question of when height standardization is required. While height may contribute to observed sexual shape dimorphism in some cases, the lack of a significant relationship with CVA scores implies that the confounding effect is method dependent. As a result, the decision to control for height should consider both the chosen scoring approach as well as the specific study objectives. To preserve as much sex variation as possible, researchers may also consider standardizing for height separately in each sex.

Our results showed that CVA outperformed regression scoring, resulting in scores that were more accurate in classifying the sexes and recreating the male–female shape axis. In contrast, the regression scores appeared to only partially capture the patterns of sex difference on which they were modelled. Male shapes captured by the regression scores possessed a distinct forehead phenotype, in which the projection of the brow extended upward to encompass the entire forehead. This forehead phenotype did not appear in either the allometry‐inclusive or allometry‐exclusive male–female shape vectors; however, we noted that it strongly resembled the shape effect from the second eigenvector. Further investigation revealed a strong and disproportionate association between the regression scores and the second principal component. The consequences of this deference to the second component, in particular, were most likely to blame for the poor modeling of sexual dimorphism that we observed. Unlike Mitteroecker et al. (2015), we did not find any easily discernible differences between the vector of CVA score shape effect and patterns of male–female shape difference. This divergence between our studies may be because our study uses three‐dimensional rather than two‐dimensional data or the larger number of principal components that we retain.

The strong and disproportionate relationship between regression scores and PC2 suggests that they are sensitive to differences in variance or scale across variables. Without prior standardization, variables with higher variances (e.g., PC2) can disproportionately influence the results, potentially skewing the model's performance. The standardization of variables by their variance is common practice within many statistical learning classification models, including support vector machines and convolutional neural networks (Hastie et al., 2009). Performed by either scaling or normalization, standardization is an important step to ensure that all variables contribute equally to the analysis. Here, we demonstrated the consequences of not standardizing in the context of sexual dimorphism of the face. Regression scores were poorer sex classifiers and less accurately reproduced the sexual dimorphism axis, with the regression score shape vector strongly resembling the second principal component. Importantly, we found that regression score performance was consistent across analyses that used principal components as well as direct input of either sparse (65‐landmarks) or dense landmark configurations (5629‐landmarks) (data not shown). In contrast, CVA rescaled variables in favor of class separation and was thus less susceptible to variance differences between variables. Other research has shown that regression scores and angles in a rescaled Mahalanobis space outperform comparable Euclidean measures (Hoskens et al., 2021; Vanneste et al., 2024). These findings emphasize the significance of standardization, demonstrating how unscaled variables can reduce regression score accuracy, whereas methods like CVA, which account for variance differences, may produce more reliable and robust model outcomes.

In our study, height was positively associated with regression scores, but not CVA scores of facial sexual dimorphism. These results are consistent with previous research that found significant differences between scores generated by regression and CVA methods. Hahn et al. (2019) investigated the link between female facial maleness and handgrip strength using both approaches. In their study, handgrip strength was only weakly correlated with regression scores and not with CVA scores, suggesting that facial maleness was not a primary indicator of physical strength in women. Alternatively, Mitteroecker et al. (2015) investigated the relationship between both scores and perceived masculinity. They found that regression scores were the better predictor of perceived masculinity, while the CVA scores showed no association. Our study may help to explain these findings: given that regression scores favor early components (or variables with greater variance and scale), it is possible that these associations reflect a relationship with some confounding variable other than sex. For example, physical strength, perceived masculinity, and other measures of social perception and reproductive success have been shown to correlate with height‐related facial cues (Batres et al., 2015; Holzleitner et al., 2014; Sell et al., 2009; Stulp et al., 2017). Therefore, the link between regression scores and these variables may reflect an association with height rather than morphological sexual dimorphism of the face. This is further supported by findings, which show a greater correlation between the allometric component of facial sexual dimorphism and perceived masculinity (Mitteroecker et al., 2015). More research is needed to understand these relationships in greater detail.

While CVA scores outperformed regression scores as classifiers and in recreating the male–female shape axis, there are some significant limitations of CVA that must be addressed. CVA is often cited for being prone to overfitting and sensitive to noise, particularly when used for significance testing between small or unbalanced groups (Menardi & Torelli, 2014; Vannatta & LaVenia, 2020). However, we did not use CVA for significant testing between males and females; rather, we focused on using CVA scores to extract meaningful shape variation associated with sex. In addition, we used parallel analysis to reduce the number of variables (greater than 20 individuals for every predictor variable) and avoided overfitting by using an age‐ and sex‐matched external holdout sample (Vannatta & LaVenia, 2020). Given these precautions, we believe that the typical limitations of CVA are not applicable.

One limitation of our study is the use of facial surface images. While facial surface scans have a strong correlation with underlying hard tissues (Young et al., 2016), they are also influenced by overlying soft tissue morphology (i.e., muscle and fat), which we are unable to fully disentangle. The relationship between hard and soft tissues varies across the face. Areas such as the forehead and nasal root likely have a stronger correlation with underlying hard tissues, whereas the cheeks likely have a greater soft tissue component (Malá et al., 2018; Qian et al., 2022). The cheeks are also more susceptible to environmental influences such as increased adiposity due to greater body weight (Švábová et al., 2024). Future facial sexual dimorphism research would benefit from an examination of both hard and soft tissue data; however, datasets are limited due to the invasive nature of the imaging modalities commonly used to obtain such information.

Our paper addresses the two most common methods of scoring sexual dimorphism used in the literature. More research is needed to understand how other methods compare, including common ratios (e.g., fWHR), non‐linear methods, and synthetic 2D image‐morphing techniques commonly used in psychology (Tiddeman & Perrett, 2002). Additionally, our study addresses a single group of white adult participants. Sex‐dependent shape differences have been shown to differ between populations depending on age (Matthews et al., 2016, 2018) and ancestry (Kleisner et al., 2021). For this reason, further work is needed to better understand how sexual dimorphism across populations interacts with diverse measurement techniques. Such comparisons are necessary to make informed decisions about which method to use and reconcile conflicting literature on the role of facial sexual dimorphism in various aspects of human health and biology.

## 5 CONCLUSIONS

We compared the two most common sexual dimorphism scoring methods used in the literature, regression scoring and CVA, which began with 3D descriptions of the face, both with and without the allometric component. Our results showed that CVA outperformed regression scoring, resulting in scores that were more accurate in classifying the sexes and recreating the male–female shape axis. The scores varied significantly between methods, yielding conflicting association results: height was significantly associated with regression scores but not CVA scores of facial sexual dimorphism. Future research is needed to explore additional measurement techniques and investigate how demographic factors, such as age and ancestry, affect facial sexual dimorphism and its quantification.

## AUTHOR CONTRIBUTIONS

CD, HH, JDA, PC, and BH conceptualized the project, including its goals and aims, and developed the research methodology. SMW collected and assembled the data for the 3DFN cohort, while CD, HH, JDA, and KC assisted with data curation and cleaning. SMW, BH, and CD secured financial support for the project. HH and CD developed the MATLAB code for data processing, curation, and quality control. CD carried out the analyses, wrote the R code, created the figures, and wrote the initial draft of the manuscript. HH, JDA, PC, and BH provided supervision and leadership, including oversight of research activity planning and execution. HH, JDA, and BH all contributed to the second draft of the manuscript. All authors contributed final revisions and approved the manuscript's final draft.

## Supporting information


Data S1.

Data S2.

Video S1.

Data S3.

Data S4.

## Data Availability

The data that support the findings of this study are openly available in 3D Facial Norms at https://www.facebase.org/, reference number DOI: https://doi.org/10.25550/VWP.
