# Supplementary material for: Measuring sexual dimorphism in human faces
Source: J Anat. 2025 Oct 16;248(5):705–18. doi: 10.1111/joa.70056 (PMC13069161; doi:10.1111/joa.70056)
Supplement: Supplementary file 4 — Data S3. [file JOA-248-705-s001.pdf]

Supplemental Tables and Figures

Table S1. Procrustes MANOVA model (Type III Sums of Squares) of height and sex on total shape in the training sample. Shape is represented by PC1-PC36. P-values were calculated from distributions generated from 10,000 resampled permutations, implemented using the procD.lm function from geomorph (Adams & Otárola-Castillo, 2013, Baken et al., 2021).

	df	SS	MS	R^2	F	Z	p-value
**Height**	1	0.01030	0.10302	0.00646	5.5893	3.6938	0.0002
**Sex**	1	0.05598	0.055982	0.03511	30.3730	7.1373	0.0001
**Residuals**	777	1.43212	0.001843	0.89814			
**Total**	779	1.59453					

Table S2. Procrustes MANOVA model (Type III Sums of Squares) of height and sex on non-allometric shape in the training sample. Shape is represented by PC1-PC36. P-values were calculated from distributions generated from 10,000 resampled permutations, implemented using the procD.lm function from geomorph (Adams & Otárola-Castillo, 2013, Baken et al., 2021).

	df	SS	MS	R^2	F	Z	p-value
**Sex**	1	0.02545	0.025449	0.0171	13.537	5.7212	0.0001
**Residuals**	777	1.46265	0.001880	0.9829			
**Total**	779	1.48810					

Supplemental Tables and Figures

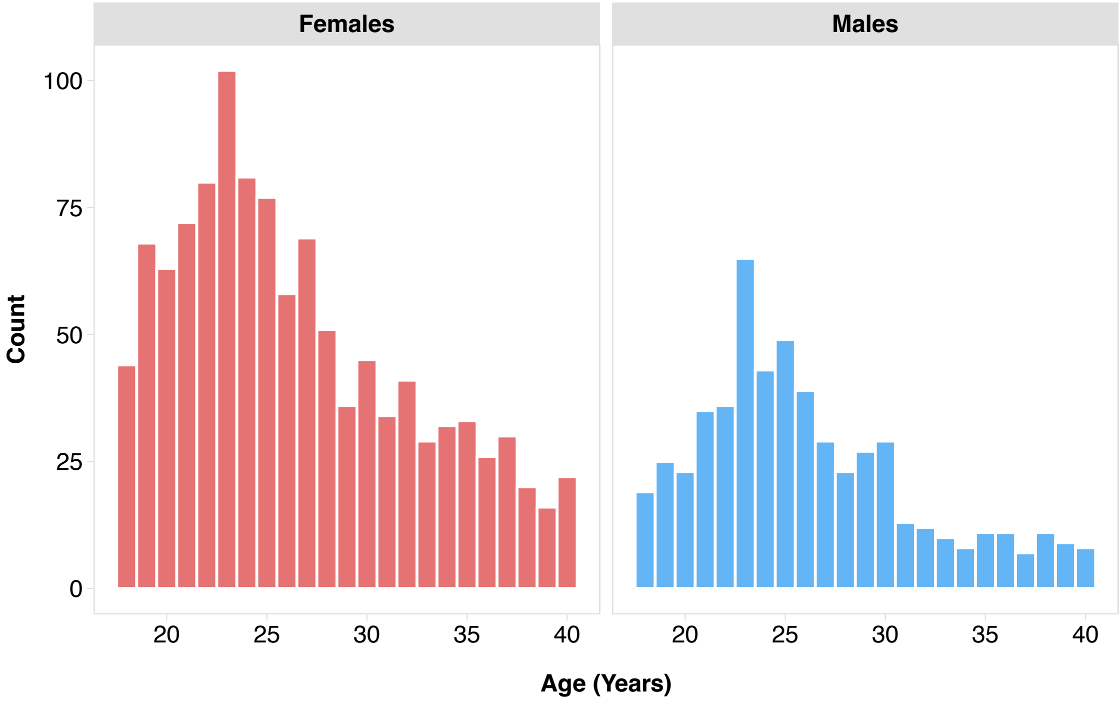

Figure S1. Distribution of ages in the 3DFN sample after data filtering, by sex. The histograms show the number of participants in each age group after filtering out participants with low-quality scans, regression outliers, height outliers, participants under the age of 18, related individuals, and cases with missing data on age, sex, height, or weight.

Supplemental Tables and Figures

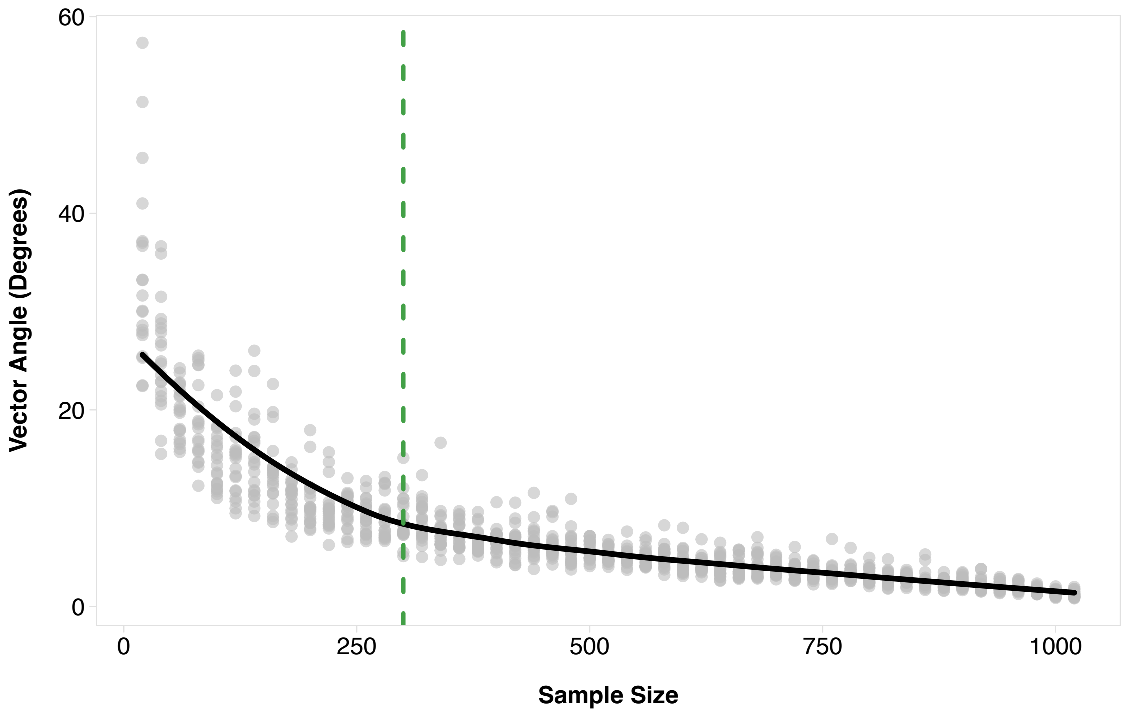

Figure S2. Relationship between sample size and the direction of male-female shape vectors. 20 permutations were performed for each sample size; each point represents the vector angle (in degrees) between the sex vector of a given iteration and the sex vector of the entire sample (N_{total} = 1,080). The LOESS curve (black line) highlights the trend, demonstrating how vector similarity changes with increasing sample size. The green dashed line represents the size of the test sample used in this study (N_{test} = 300). Landmark configurations were aligned with General Procrustes analysis (Schlager, 2017) prior to the permutation testing.

Supplemental Tables and Figures

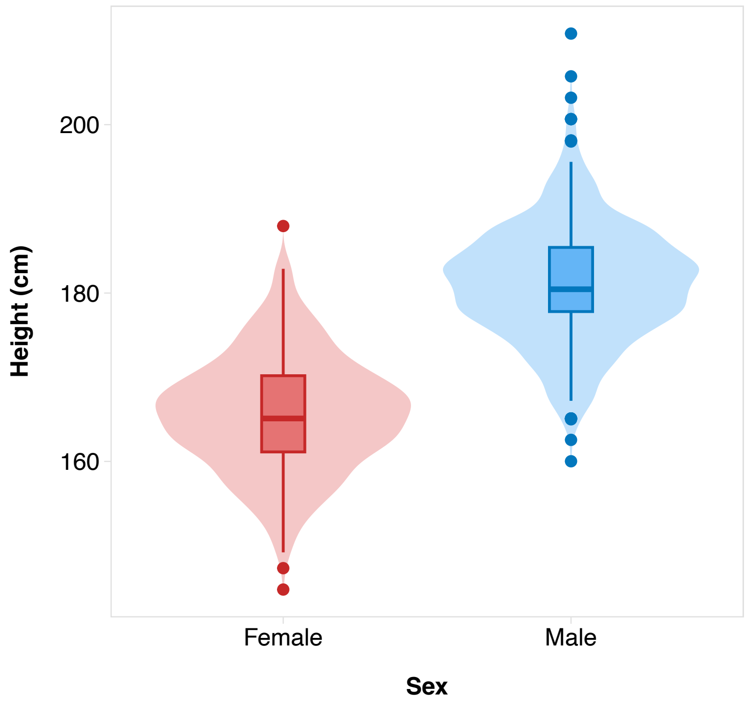

Figure S3. Violin and boxplot of the height distribution in the training sample, stratified by sex. The violin plot depicts the density of participant heights, with a boxplot overlay showing the interquartile range (box limits) and median height (bold line). Whiskers extend to 1.5 times the interquartile range, with participants outside of this range represented by individual data points.

Supplemental Tables and Figures

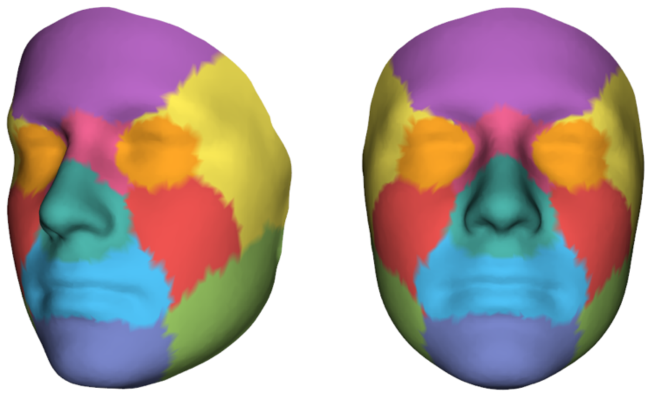

Figure S4. Data-driven facial segmentation using k-means clustering. 3D morphs depict the locations of nine bilaterally symmetric facial segments, such as the frontal (purple), temporal and zygomatic (yellow), orbital (orange), infraorbital (red), nasal root (pink), nasal (turquoise), oral (blue), buccal and parotid (green), and mental (indigo) regions.

Supplemental Tables and Figures

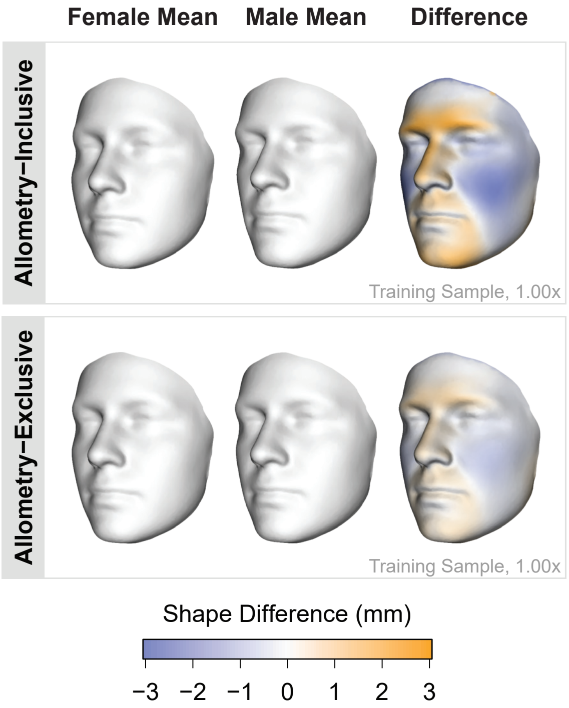

Figure S5. Unscaled 3D morphs showing the effects of sex on shape in the training sample, both with and without the allometric component. Female and male average morphs from the training sample are shown alongside heatmaps indicating the differences between them (i.e., how the male morph differs from the female morph).

Supplemental Tables and Figures

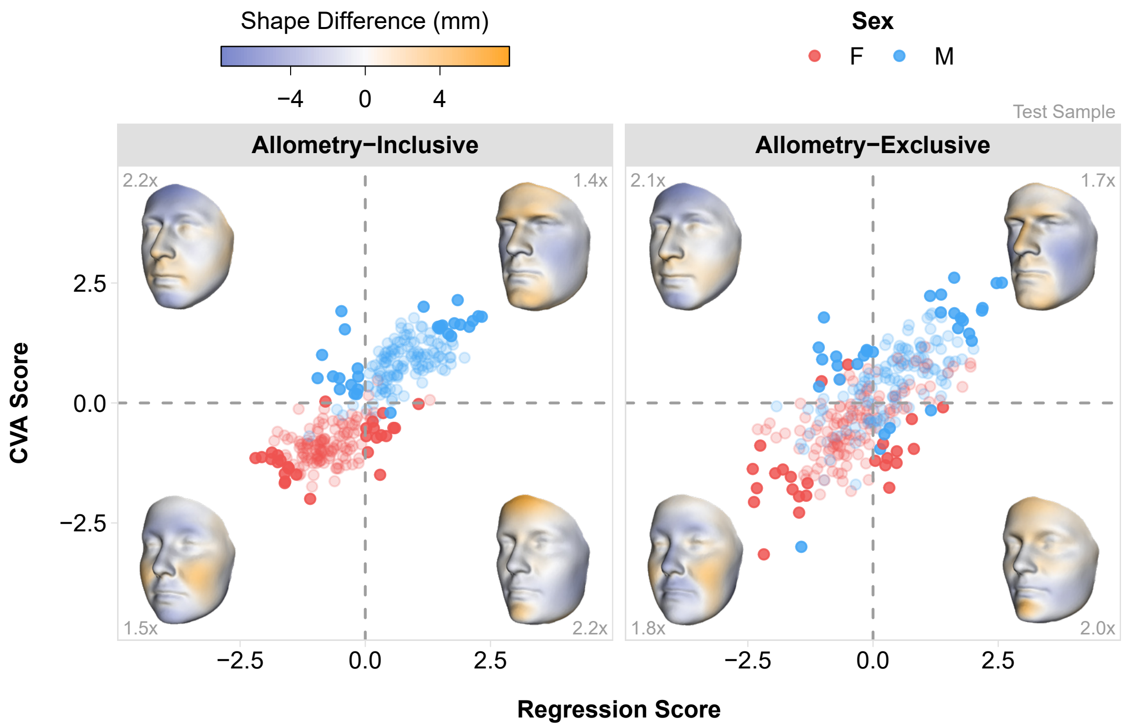

Figure S6. Comparison of shapes scored as closer to the male mean (>0) in one method and closer to the female mean (<0) in another, and vice versa. 3D scaled heatmaps of the average faces from the test sample are displayed alongside their corresponding sampled subsets (N=14), with the scale representing the shape difference from the sample mean.

Supplemental Tables and Figures

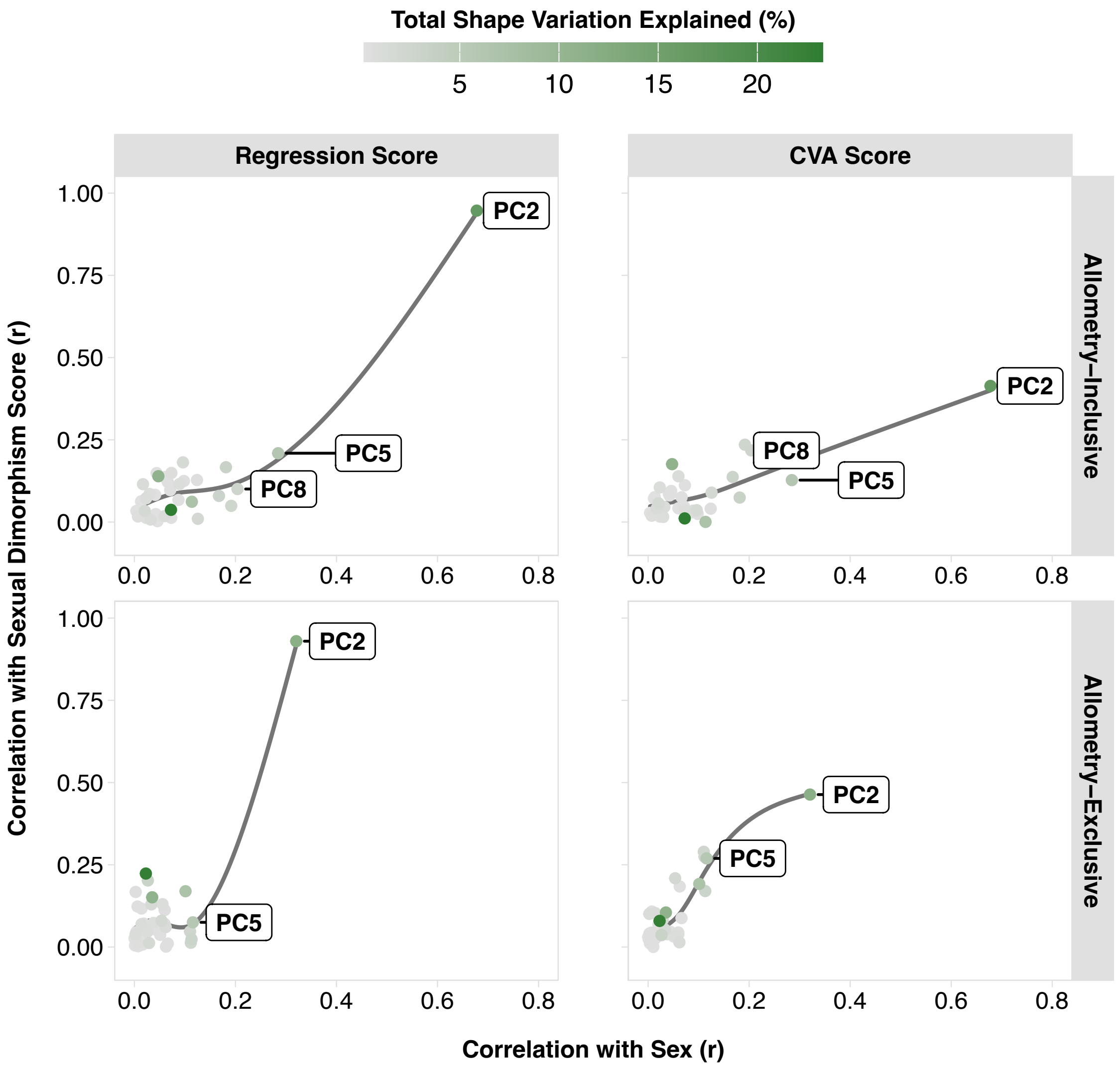

Figure S7. Relationship between principal components, sex, and sexual dimorphism scores. Trend lines are fitted with a gamma function; points are colored based on the percentage of total shape variation (prior to removal of allometric variation) explained by the principal component in the training sample.
