# Supplementary material for: Measuring sexual dimorphism in human faces
Source: J Anat. 2025 Oct 16;248(5):705–18. doi: 10.1111/joa.70056 (PMC13069161; doi:10.1111/joa.70056)
Supplement: Supplementary file 5 — Data S4. [file JOA-248-705-s005.docx]

**Video S1. Allometry-inclusive facial sexual dimorphism.** GIF showing the difference between scaled 3D morphs of sex on shape in the training sample, with the allometric component included (3.58x). Exaggerated male and female morphs from the training sample are shown alongside heatmaps indicating their differences from the mean (i.e., the midpoint between male and female shapes).
